# Algorithmic performance appraisal intensity in hospitals: implications for professional autonomy and innovative work behavior

**DOI:** 10.3389/fpsyg.2026.1886547

**Published:** 2026-07-29

**Authors:** Qing Yang, Shuang Li, Kai Wang, Xin Chang, Shengya Cao, Guihua Zhang

**Affiliations:** 1 China University of Mining and Technology, Xuzhou, China; 2 Xuzhou Third People’s Hospital, Xuzhou, China

**Keywords:** algorithmic HRM, algorithmic performance appraisal, algorithmic transparency, innovative work behavior, professional autonomy

## Abstract

**Introduction:**

Hospitals increasingly use algorithmic systems to monitor, compare, score, and appraise employees. Although such systems may improve consistency and efficiency, intensive use may constrain medical professionals’ autonomy and their innovative behavior. This study examined the relationships among algorithmic performance appraisal intensity, perceived professional autonomy, and innovative work behavior, and tested whether algorithmic transparency moderates these relationships.

**Methods:**

Three studies were conducted. Study 1 was a between-subjects vignette experiment (*N* = 240) comparing high- and low-intensity appraisal. Study 2 used a two-wave survey of medical professionals (*N* = 287) to test mediation. Study 3 used a three-wave, multisource design (*N* = 273), with supervisors rating innovative work behavior, to test moderation and moderated mediation.

**Results:**

In Study 1, high appraisal intensity reduced perceived professional autonomy (t(237.92) = 6.66, *p* < 0.001, Cohen’s d = 0.86). In Study 2, professional autonomy positively predicted innovative work behavior (b = 0.192, *p* < 0.001), but appraisal intensity did not significantly predict autonomy (b = −0.133, *p* = 0.055), and the indirect effect was not significant. In Study 3, appraisal intensity was negatively associated with autonomy (b = −0.189, *p* = 0.002), autonomy positively predicted innovative work behavior (b = 0.126, *p* = 0.028), and transparency was positively associated with autonomy (b = 0.150, *p* = 0.011). However, neither the interaction between appraisal intensity and transparency (b = 0.046, *p* = 0.300) nor the index of moderated mediation was significant.

**Discussion:**

Across the three studies, intensive algorithmic appraisal showed inconsistent but generally autonomy-constraining relationships, whereas professional autonomy consistently supported innovative work behavior. Transparency was positively associated with autonomy but did not reliably buffer the negative effects of appraisal intensity. Hospitals should retain contextual human judgment and treat transparency as a supportive design feature rather than assume that it offsets intensive algorithmic control.

## 1 Introduction

The digital transformation of hospitals increasingly extends beyond electronic medical records and clinical decision-support systems to internal workforce management. Hospitals use digital platforms to record employees’ work activities, compare individual performance indicators, generate scores and feedback, and support decisions concerning pay, promotion, and access to organizational resources. These practices represent algorithmic performance appraisal, an employee-level HRM process in which digital systems and rule-based algorithms support the monitoring, comparison, and appraisal of individual work performance.

In China, this development is reinforced by the national performance-monitoring system for public hospitals, which emphasizes standardized data collection, indicator calculation, comparison, and feedback (National Health Commission of the People’s Republic of China, 2025). However, the underlying issue is not unique to China. European case studies have documented algorithmic management in regular healthcare workplaces (Rani et al., 2024), while an OECD employer survey covering France, Germany, Italy, Japan, Spain, and the United States shows that software-assisted monitoring and evaluation are increasingly relevant across national settings (Milanez et al., 2025). These developments raise a shared question: how can professional organizations use data-driven appraisal without undermining employees’ professional judgment and autonomy?

Performance appraisal translates information about individual performance into feedback, development, rewards, promotion, and other personnel decisions (DeNisi and Murphy, 2017; Schleicher et al., 2018). Algorithmic HRM extends this process by transferring some monitoring, comparison, scoring, and recommendation functions from human managers to data-driven systems (Tambe et al., 2019). When algorithmically generated scores influence pay, promotion, or resource allocation, appraisal systems operate not only as informational mechanisms but also as forms of behavioral and motivational control. Algorithms can reshape managerial authority by continuously directing and evaluating employee behavior (Kellogg et al., 2020). From a self-determination theory perspective, highly controlling appraisal arrangements may orient employees toward external indicators and contingent consequences, thereby weakening autonomy and self-endorsed motivation (Gagné et al., 2022).

This issue is especially important in medical work, where professional knowledge, contextual judgment, and discretion are central. Medical professionals often combine clinical, research, teaching, and discipline-development responsibilities, and their value cannot always be reduced to short-term measurable outputs (Awowale, 2017). Professional autonomy is related not only to job control but also to healthcare professionals’ ability to respond proactively, adapt to changing demands, and generate innovative solutions (Pursio et al., 2024; Kessel et al., 2012; Pederson and Slowiak, 2026). Prior studies have linked professional autonomy, autonomy-supportive leadership, professional respect, and empowering climates to work performance and innovative behavior among healthcare workers (van Dorssen-Boog et al., 2022; Lu et al., 2025). Algorithmic appraisal may therefore create tension between standardized evaluation and the professional discretion required in hospital work.

Algorithmic management has moved from platform work into conventional organizations and professional settings (Meijerink and Bondarouk, 2023; Sullivan, 2024; Keegan and Meijerink, 2025). Hospitals are particularly important because they face strong performance pressures while depending on human-centered judgment (Monteil et al., 2025). Algorithmic systems can improve information-processing efficiency and consistency, but may also weaken employees’ sense of control and motivation (Bahangulu and Owusu-Berko, 2025; Li et al., 2024). Even when algorithmic evaluation appears objective, employees may experience it as lacking respectful treatment or individualized consideration and may feel reduced to data objects (Chun et al., 2024). The key issue is therefore not only whether algorithmic appraisal is accurate, but also how it changes employees’ perceptions of professional agency and autonomy.

Professional autonomy may also help explain innovative work behavior in hospitals. Innovative work behavior includes generating ideas, identifying problems, promoting new approaches, and implementing useful changes (Scott and Bruce, 1994). In hospitals, it involves improvements to clinical workflows, patient services, interdisciplinary coordination, and work-related problem solving while remaining consistent with professional and patient-safety requirements. Such behavior is especially important when established procedures cannot fully accommodate clinical complexity (Kessel et al., 2012). Existing healthcare research has linked autonomy and empowering work environments to innovative behavior (Baig et al., 2022; Lu et al., 2025), but less is known about whether employee-level algorithmic appraisal shapes autonomy and, in turn, innovation.

The role of algorithmic transparency also remains uncertain. Medical AI research has focused mainly on clinical decision support, explainability, trust, safety, and accountability (Labkoff et al., 2024; Goktas and Grzybowski, 2025; Jones et al., 2023). Clinical acceptance of AI depends substantially on transparency, auditability, and human oversight (Abbas et al., 2025; Tun et al., 2025), yet less attention has been paid to transparency in internal hospital performance appraisal. It remains unclear whether understanding data sources, evaluation standards, scoring logic, and feedback mechanisms is associated with professional autonomy and innovative work behavior, or whether it changes how appraisal intensity is experienced (Donia, 2025).

Three gaps remain. First, research on algorithmic management has focused mainly on platform work and general organizational settings, leaving individual-level algorithmic performance appraisal in hospitals underexamined. Second, it remains unclear whether and how appraisal intensity is related to medical professionals’ innovative work behavior through perceived professional autonomy. Existing healthcare research has emphasized leadership, organizational climate, and job resources, but has paid less attention to appraisal systems as antecedents of autonomy and innovation. Third, the role of algorithmic transparency in hospital performance appraisal remains insufficiently understood, including whether it is related to autonomy and innovative work behavior and whether it conditions the relationship between appraisal intensity and autonomy.

To address these gaps, this study conceptualizes algorithmic performance appraisal intensity as an individual-level performance management characteristic capturing the extent to which hospitals rely on digital systems and rule-based algorithms to monitor, compare, score, and appraise individual medical professionals and inform personnel decisions. It examines the relationships among appraisal intensity, perceived professional autonomy, and innovative work behavior, the explanatory role of professional autonomy, and the role of algorithmic transparency.

This study makes three contributions. First, it extends research on performance management and algorithmic HRM to individual-level algorithmic performance appraisal in hospitals, a professional setting in which standardized evaluation must coexist with contextual judgment and professional discretion. Second, it examines professional autonomy as an important correlate of innovative work behavior among medical professionals and provides evidence that employees who perceive greater professional discretion are more likely to engage in innovation. Third, it clarifies the role of algorithmic transparency by distinguishing its positive association with professional autonomy from its unsupported moderating role. This distinction provides a more cautious understanding of transparency in algorithmic appraisal systems.

## 2 Theoretical background and hypotheses

### 2.1 Algorithmic performance appraisal intensity and perceived professional autonomy

Algorithmic performance appraisal uses digital systems and rule-based algorithms to collect, compare, and interpret individual employees’ performance data and generate scores, rankings, and feedback that may inform pay, promotion, development, and resource allocation decisions (DeNisi and Murphy, 2017; Schleicher et al., 2018; Parent-Rocheleau and Parker, 2022; Tambe et al., 2019). In this study, algorithmic performance appraisal intensity refers to the extent to which hospitals rely on these system-generated processes and outputs when evaluating individual medical professionals.

The focal issue is the degree of algorithmic involvement in appraisal rather than the mere use of digital tools. Under high intensity, employees’ work is continuously tracked and compared, and automated scores, rankings, or recommendations substantially influence appraisal outcomes. Under low intensity, digital systems primarily record and summarize information, while supervisors retain greater responsibility for contextual interpretation and final appraisal decisions (Li et al., 2025; Osonuga et al., 2025).

Algorithmic performance appraisal intensity is conceptually distinct from general performance pressure. Performance pressure concerns employees’ perceived demands to meet difficult targets or avoid unfavorable consequences, regardless of whether appraisal is conducted by human managers or digital systems. By contrast, algorithmic appraisal intensity concerns the extent to which evaluative authority is embedded in continuous data collection, automated comparison, standardized scoring rules, and system-generated outputs. A performance system may therefore be highly demanding but primarily human-led, or highly algorithmic without imposing higher targets or workloads. The proposed autonomy effect arises from the transfer of evaluative discretion to standardized system rules, rather than simply from stronger performance demands.

Although algorithmic involvement can improve consistency and information-processing efficiency, it may also reshape how managerial control is exercised and experienced (Meijerink and Bondarouk, 2023; Kellogg et al., 2020; Sullivan, 2024). When system-generated evaluations influence consequential personnel decisions, employees may become increasingly oriented toward measurable indicators and externally determined outcomes. From a self-determination theory perspective, such appraisal may be experienced as controlling when employees have limited opportunities to interpret, question, or contextualize system-generated results, thereby weakening autonomy and self-endorsed motivation (Gagné et al., 2022).

This concern is particularly relevant in hospitals because medical work requires professional experience, patient-specific judgment, and decision-making under complex and uncertain conditions (Afonso and Ferreira, 2026). Professional autonomy is grounded in competence, independent judgment, and discretion, but it is also shaped by organizational systems and managerial practices (Mrayyan et al., 2024; Rouhi-Balasi et al., 2020; Yuk and Yu, 2023; Pursio et al., 2024). When appraisal systems emphasize quantifiable indicators such as service volume, medical record quality, patient evaluations, research output, and efficiency, medical professionals may have fewer opportunities to explain contextual circumstances or incorporate contributions that are difficult to quantify.

Algorithmic performance appraisal may therefore affect not only work visibility but also who defines good performance and according to what logic (van Zoonen et al., 2025). If predefined indicators and automated comparisons increasingly determine how professional work is interpreted, medical professionals may adjust their behavior around measurable outputs rather than exercise contextual judgment. Perceived algorithmic control has also been associated with reduced autonomy, frustration, and stress (Siegel et al., 2022). This issue is especially important for medical professionals whose value also lies in complex problem solving, knowledge integration, and continuous improvement that may not be fully captured by short-term metrics (Zhou et al., 2025). Accordingly, we propose:

*H1*: Algorithmic performance appraisal intensity negatively affects medical professionals’ perceived professional autonomy.

### 2.2 Perceived professional autonomy and innovative work behavior

Perceived professional autonomy may promote innovative work behavior because it provides the psychological basis for individuals to actively change existing practices (Gagné et al., 2022). Innovative work behavior extends beyond generating new ideas to include identifying problems and promoting and implementing useful changes (Scott and Bruce, 1994). In hospitals, it refers to medical professionals’ efforts to develop and apply new approaches to clinical workflows, patient services, interdisciplinary coordination, and work-related problem solving, while remaining consistent with professional standards and patient-safety requirements. Such behavior is particularly important when established procedures cannot fully accommodate the complexity of clinical practice (Kessel et al., 2012). Medical professionals therefore need a certain degree of judgment and action space within the institutional framework. According to self-determination theory, autonomy is not a peripheral feature of work. It is an important source of high quality motivation (Van Yperen et al., 2016). When individuals feel that they can make choices based on professional knowledge and guide their work process, their behavior is more likely to be driven by internal endorsement rather than external compliance.

In hospitals, perceived professional autonomy is important because medical innovation is rarely an extra task separated from daily work. It is often embedded in diagnosis, treatment, nursing, coordination, and continuous service improvement. Only when medical professionals believe that they have the right to make judgments according to the situation are they more likely to depart from routine paths and try new communication methods, service processes, or problem solving approaches. Baig et al. (2022), using a nurse sample, found that job autonomy not only directly affects innovative work behavior, but also indirectly shapes innovation through cognitive appraisal and work engagement. In other words, higher autonomy makes individuals more likely to interpret work demands as manageable challenges rather than as external pressures that require passive obedience. Similarly, Palumbo (2021) found that positive work experiences and empowering organizational environments increase healthcare professionals’ willingness to engage in innovation-oriented behavior. This further indicates that innovation does not arise from capability alone. It also depends on whether individuals have sufficient agency and room for action.

More importantly, in healthcare organizations, perceived professional autonomy is not only associated with innovative work behavior. It may also be a proximal condition that enables innovation to occur consistently. Recent research provides direct evidence for this point. Lu et al. (2025) found that clinical nurses’ professional autonomy is positively associated with innovative behavior and mediates the relationship between empowering management and innovation. This finding suggests that innovative behavior is not a mechanical response to managerial support. It is grounded in individuals’ subjective judgment about whether they can still guide their own professional work. When perceived professional autonomy is strong, medical professionals are more likely to identify opportunities for improvement and accept the uncertainty associated with innovation. In contrast, when they feel that their decision making space is restricted, they may prefer to maintain existing practices even if they have the ability to innovate. Therefore, this study argues that higher perceived professional autonomy leads to stronger innovative work behavior among medical professionals. Accordingly, the following hypothesis is proposed:

*H2*: Perceived professional autonomy positively affects medical professionals’ innovative work behavior.

### 2.3 The mediating role of perceived professional autonomy

Perceived professional autonomy may mediate the relationship between algorithmic performance appraisal intensity and innovative work behavior because these two variables operate at different levels of the same process. Algorithmic performance appraisal intensity is first an organizational governance arrangement. It determines how work is recorded, compared, and evaluated (Wang, 2026). Innovative work behavior, by contrast, is an individual behavior that involves discretion, extra effort, and uncertainty (Goepel et al., 2012). For medical professionals, innovation often depends on role flexibility, knowledge integration, and the ability to implement new solutions. These conditions require individuals to retain a certain degree of discretionary space within the existing institutional framework (Kessel et al., 2012).

Accordingly, algorithmic performance appraisal intensity is unlikely to directly determine whether innovative behavior increases or decreases. It is more likely to first change medical professionals’ subjective judgment about their control over professional work, and then influence whether they are willing to depart from routine practices, take improvement related risks, and engage in innovation. According to self-determination theory, the effects of external control structures on behavior do not occur in a single step. Instead, they are transmitted through the satisfaction of autonomy needs, the quality of self-regulation, and work engagement (Gagné et al., 2022). Job autonomy is positively related to innovative work behavior and may also affect innovation through more proximal psychological processes, such as cognitive appraisal, work engagement, and burnout (Pederson and Slowiak, 2026). This suggests that autonomy is not a secondary work experience. It is an important psychological mechanism that connects work systems with innovative behavior.

This mediating logic is particularly relevant in hospitals. Medical professional work depends heavily on professional judgment and responsibility. Changes in performance evaluation methods are therefore likely to first affect the core question of whether medical professionals can still guide their own work, before influencing subsequent behavior. Existing healthcare research has shown that professional autonomy mediates the relationship between empowering management and innovative behavior. This indicates that organizational conditions often lead to innovation because they first change medical workers’ perception of autonomous decision-making space (Lu et al., 2025). Similarly, the positive effects of job autonomy on healthcare workers are transmitted through more proximal psychological mechanisms, rather than appearing only as simple direct effects (van Dorssen-Boog et al., 2020). Following this logic, algorithmic performance appraisal intensity, as a more upstream form of digital governance, is likely to inhibit medical professionals’ innovative work behavior by reducing their perceived professional autonomy. Therefore, this study proposes the following hypothesis:

*H3*: Perceived professional autonomy mediates the relationship between algorithmic performance appraisal intensity and medical professionals’ innovative work behavior.

### 2.4 The moderating role of algorithmic transparency

Although algorithmic performance appraisal intensity may weaken medical professionals’ perceived professional autonomy, this effect may not be equally strong in all situations. A key condition is how individuals understand the system. Algorithmic transparency matters not because it removes the controlling nature of performance evaluation, but because it shapes how this control is interpreted (Lepri et al., 2018). When the sources of indicators, evaluation criteria, calculation logic, and feedback mechanisms are relatively clear, employees are more likely to view the system as an identifiable and predictable managerial arrangement (Edwards et al., 2024). Prior studies have shown that algorithmic transparency is closely related to procedural fairness, trust, and proactive behavior (Bitzer et al., 2023; Lee et al., 2019). In performance management settings, transparent algorithmic systems are more likely to motivate employees to improve their performance and engage in proactive work behavior. In contrast, opaque algorithmic evaluation is more likely to generate uncertainty and negative reactions (Cai and Wang, 2024; Ochmann et al., 2024; Jabagi et al., 2025).

At the same time, the negative effects of algorithms are not determined only by the strength of control. They also depend on whether employees can understand and anticipate how the system operates. When algorithmic performance appraisal is intense but its logic is transparent, employees may be more likely to interpret it as a clear performance rule. When the evaluation logic is opaque, however, employees may experience the system as an intrusion of external technical logic into their work space, which may further intensify the loss of autonomy (Hu et al., 2024). This logic is especially important in hospitals. Medical professional work depends strongly on professional judgment, and professional autonomy is a core basis on which individuals judge whether their professional status is preserved (Hoogland and Jochemsen, 2000). If an algorithmic system clearly explains its evaluation basis and allows medical professionals to understand what data are included, why they are included, and how results are generated, algorithmic intervention may still create rules and constraints, but it may not necessarily be interpreted as a direct replacement for professional judgment. By contrast, under low transparency, stronger algorithmic evaluation may make medical professionals feel that their work is being reduced to several calculable indicators, thereby compressing their space for professional discretion. Therefore, this study proposes the following hypothesis:

*H4*: Algorithmic transparency weakens the negative effect of algorithmic performance appraisal intensity on medical professionals’ perceived professional autonomy.

The above logic can be further extended to the overall indirect effect. As discussed earlier, algorithmic performance appraisal intensity may not directly determine innovative work behavior. Rather, it may influence medical professionals’ willingness to depart from routine practices, take improvement related risks, and engage in innovation by changing their perceived professional autonomy. Therefore, if algorithmic transparency buffers the negative effect of algorithmic performance appraisal intensity on perceived professional autonomy, this buffering effect should also extend to the subsequent path leading to innovative work behavior (Barnes, 2025). Algorithmic transparency not only affects employees’ judgments of fairness and acceptability, but also shapes behavioral outcomes such as proactive work behavior, resistance, and deviant behavior (Cai and Wang, 2024).

For medical professionals, acceptance of algorithmic systems depends largely on whether they can understand the basis of system generated recommendations or evaluations, and whether they still retain sufficient space for judgment and correction (Tun et al., 2025). When such understanding and space are preserved, the threat of technical systems to professional autonomy may be reduced. As a result, the behavioral inhibition caused by damaged autonomy may also become weaker (Funer and Wiesing, 2024; Nouis et al., 2025). Therefore, under high transparency, algorithmic performance appraisal intensity may still affect medical professionals through standardization and data driven logic, but its negative indirect effect on innovative work behavior through perceived professional autonomy should be weaker. Under low transparency, this negative indirect effect is more likely to be amplified. Accordingly, the following hypothesis is proposed:

*H5*: Algorithmic transparency moderates the indirect effect of algorithmic performance appraisal intensity on innovative work behavior through perceived professional autonomy, such that this negative indirect effect is weaker under high algorithmic transparency.

**Figure 1:**
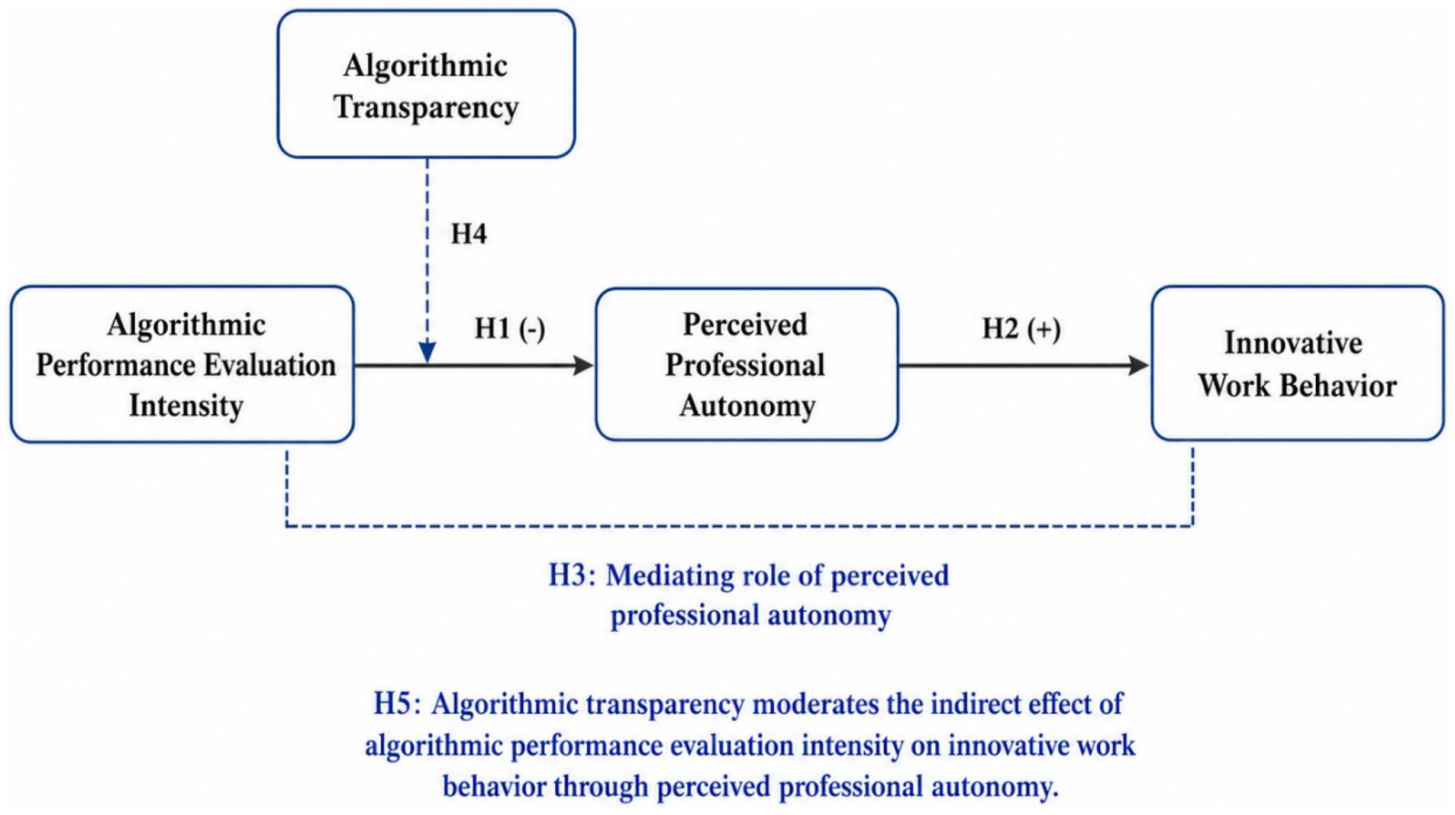
Research model.

The research model is shown in Figure 1.

## 3 Methods and results

### 3.1 Study 1

#### 3.1.1 Research design and sample

Study 1 used a vignette experiment to test the causal effect of algorithmic performance appraisal intensity on medical professionals’ perceived professional autonomy. We adopted a single factor between subjects design and randomly assigned participants to either a high or low algorithmic performance appraisal intensity condition. The two scenarios were identical in hospital type, job responsibilities, work tasks, and the purpose of performance evaluation. Only the degree of algorithmic involvement in performance evaluation was varied.

Participants were individuals with medical work experience or medical training backgrounds, including physicians, nurses, medical technicians, and medical graduate students with clinical internship or standardized residency training experience. A total of 260 responses were initially collected. After excluding participants who failed the attention check, scenario comprehension check, or showed abnormal response patterns, 240 valid responses were retained. Among them, 126 participants were assigned to the low intensity condition and 114 to the high intensity condition. No obvious imbalance was found across gender, job type, age, or work experience, suggesting that random assignment was generally effective.

#### 3.1.2 Experimental materials, measures, and analytical strategy

The vignette described a tertiary general hospital upgrading its individual employee performance appraisal system. In both conditions, the appraisal target was the focal medical professional rather than the hospital or department. In the high algorithmic performance appraisal intensity condition, the hospital used a digital system to continuously collect and compare the focal employee’s data on service volume, work efficiency, quality indicators, patient evaluations, and research-related performance. The system automatically generated individual monthly scores, rankings, and feedback, which were used as references for the employee’s performance pay, promotion, and access to organizational resources. In the low algorithmic performance appraisal intensity condition, the focal employee’s performance was primarily appraised by department leaders based on daily work, periodic summaries, contextual information, and managerial discussion. The digital system only recorded information and provided auxiliary statistics, while human supervisors retained primary appraisal discretion. The manipulation represented two levels of reliance on algorithmically generated appraisal outputs commonly found in hospital performance management. In the high-intensity condition, system-generated scores, rankings, and feedback formed the primary basis of appraisal. In the low-intensity condition, digital systems provided supporting information, while supervisors retained greater responsibility for contextual interpretation and appraisal judgment. The experimental contrast therefore captured a shift in the relative weight assigned to algorithmic outputs and human judgment. The two scenarios did not differ in managerial support, workload, fairness climate, or transparency.

Participants first read the informed consent statement and confirmed their medical learning or work background. They were then randomly assigned to one of the two scenarios. After reading the scenario, they completed items measuring scenario immersion, manipulation checks, perceived professional autonomy, and demographic information. Attention check and scenario comprehension items were included to identify low quality responses.

The manipulation check for algorithmic performance appraisal intensity included three items assessing perceived reliance on system generated results, data driven analysis, and continuous monitoring. Perceived professional autonomy was measured using items adapted from Breaugh’s work autonomy scales and the Work Design Questionnaire (Breaugh, 1985, 1999; Morgeson and Humphrey, 2006). The items captured perceived judgment authority, decision making space, and control over professional work. All items used a seven point Likert scale, ranging from 1, strongly disagree, to 7, strongly agree.

Because the independent variable was experimentally manipulated, we did not estimate a full structural measurement model. Instead, we examined manipulation validity, internal consistency, and between group differences. Cronbach’s alpha values were reported for the manipulation check and perceived professional autonomy. Welch independent samples t tests were used to compare the two conditions. An analysis of covariance was also conducted after controlling for gender, age, job type, and work experience. All statistical tests were two tailed. Gender, age, occupational role, and work experience were included as covariates. Occupational role was dummy coded, and the complete control-adjusted model is reported in Supplementary Table S1.

#### 3.1.3 Results

The manipulation check and perceived professional autonomy scale showed good internal consistency, with Cronbach’s alpha values of 0.862 and 0.872, respectively.

As shown in Table 1, the manipulation was successful. Participants in the high intensity condition reported significantly higher manipulation check scores than those in the low intensity condition, *t*(229.38) = 11.10, *p* < 0.001. We then tested the main effect. Participants in the high intensity condition reported significantly lower perceived professional autonomy than those in the low intensity condition, *t*(237.92) = 6.66, *p* < 0.001, Cohen’s *d* = 0.86. Thus, H1 was supported in Study 1.

**Table 1:** Manipulation check and main effect test in Study 1.

Variable	Low algorithmic performance appraisal intensity *M* (SD)	High algorithmic performance appraisal intensity *M* (SD)	*t*	*p*	Cronbach’s *α*	Cohen’s *d*
Manipulation check	3.90 (1.29)	5.59 (1.06)	11.10	<0.001	0.862	1.43
Perceived professional autonomy	4.54 (1.31)	3.43 (1.28)	6.66	<0.001	0.872	0.86

The effect remained significant after controlling for gender, age, occupational role, and work experience, *b* = −1.110, SE = 0.168, *t* = −6.620, *p* < 0.001. The inclusion of these covariates did not alter the conclusion. Therefore, H1 was supported in Study 1. The complete control-adjusted model is reported in Supplementary Table S1.

Overall, Study 1 showed that medical professionals reported lower perceived professional autonomy under an algorithm-dominant appraisal arrangement than under a human-led arrangement supported by digital information. Study 2 examined whether this relationship also emerges in hospital field data and whether professional autonomy links appraisal intensity to innovative work behavior.

### 3.2 Study 2

#### 3.2.1 Research design and sample

Study 2 examined the mediating role of perceived professional autonomy between algorithmic performance appraisal intensity and innovative work behavior in real hospital settings. Building on the causal evidence from Study 1, we adopted a two stage survey design to separate the measurement of the antecedent, mediator, and outcome variables over time, thereby reducing common method bias.

The sample consisted of medical professionals from several general and specialized hospitals, including physicians, nurses, and medical technicians. At the first stage, 340 questionnaires were distributed, and 312 valid responses were obtained after invalid responses were removed. About 3 weeks later, the second stage questionnaire was distributed to these valid respondents. After anonymous code matching, 287 valid matched responses were retained. At T1, participants reported algorithmic performance appraisal intensity and control variables. At T2, they reported perceived professional autonomy and innovative work behavior. The sample varied in gender, job type, age, work experience, educational level, professional title, and hospital type.

#### 3.2.2 Measures and analytical strategy

Study 2 measured algorithmic performance appraisal intensity, perceived professional autonomy, innovative work behavior, and control variables. All items used a seven point Likert scale, ranging from 1, strongly disagree, to 7, strongly agree.

Algorithmic performance appraisal intensity was measured using a five-item scale developed for this study. The items were not adopted from a single existing instrument but were developed from prior research on performance appraisal, algorithmic HRM, and algorithmic control. This literature identifies continuous digital monitoring, automated comparison, rule-based scoring, system-generated feedback, and reliance on algorithmic outputs in personnel decisions as central features of algorithmic appraisal (Tambe et al., 2019; Kellogg et al., 2020; Parent-Rocheleau and Parker, 2022; Meijerink and Bondarouk, 2023). Before responding, participants were instructed to consider how their own work performance was monitored and appraised by the hospital. The five items assessed the extent to which the hospital continuously tracked individual performance data, automatically compared performance information, generated evaluations according to predefined rules, produced system-based scores or feedback, and relied on these outputs in the employee’s final performance appraisal. The complete item wording is provided in Supplementary Table S4.

Perceived professional autonomy was measured using items adapted from Breaugh’s work autonomy scales and the Work Design Questionnaire (Breaugh, 1985, 1999; Morgeson and Humphrey, 2006). The items captured medical professionals’ perceived judgment authority, decision making space, and agency in professional work.

Innovative work behavior was measured using items adapted from Scott and Bruce’s (1994) scale. The items were adapted to capture medical professionals’ generation, promotion, and implementation of new ideas concerning workflows, patient services, interdisciplinary coordination, and work-related problem solving.

Gender, age, job type, work experience, educational level, professional title, and hospital type were included as control variables. Before hypothesis testing, we assessed reliability, convergent validity, discriminant validity, and common method bias. Cronbach’s alpha, composite reliability, AVE, confirmatory factor analysis, Harman’s single factor test, and model comparison were used. Descriptive statistics and correlations were then examined, followed by regression analysis and bootstrap tests of the indirect effect. All statistical tests were two tailed.

#### 3.2.3 Measurement model

The measurement results showed good reliability and convergent validity. Cronbach’s alpha values for algorithmic performance appraisal intensity, perceived professional autonomy, and innovative work behavior were 0.848, 0.819, and 0.857, respectively. Composite reliability values were 0.873, 0.838, and 0.882, and AVE values were 0.581, 0.563, and 0.556. All standardized factor loadings were significant and ranged from 0.64 to 0.82.

Confirmatory factor analysis supported the discriminant validity of the three constructs. The three factor model fit the data well, *χ*^2^(87) = 103.99, CFI = 0.990, TLI = 0.988, RMSEA = 0.026, SRMR = 0.034. By contrast, the two factor model combining algorithmic performance appraisal intensity and perceived professional autonomy showed poorer fit, *χ*^2^(89) = 542.82, CFI = 0.729, TLI = 0.680, RMSEA = 0.134, SRMR = 0.140. The two factor model combining perceived professional autonomy and innovative work behavior also showed poor fit, *χ*^2^(89) = 504.74, CFI = 0.752, TLI = 0.707, RMSEA = 0.128, SRMR = 0.120. The one factor model showed the worst fit, *χ*^2^(90) = 1039.45, CFI = 0.433, TLI = 0.338, RMSEA = 0.192, SRMR = 0.192. In addition, the square root of AVE for each construct exceeded its correlations with other constructs.

Common method bias was also assessed. Harman’s single factor test showed that the first unrotated factor explained 27.27% of the total variance, and the one factor model fit the data much worse than the three factor model. These results suggest that common method bias was unlikely to seriously affect the findings.

As shown in Table 2, perceived professional autonomy was positively related to innovative work behavior, *r* = 0.226, *p* < 0.001. algorithmic performance appraisal intensity was not significantly related to perceived professional autonomy, *r* = −0.111, *p* = 0.060. Its correlation with innovative work behavior was not significant, *r* = −0.057, *p* = 0.335. Overall, the results suggest a stable positive relationship between perceived professional autonomy and innovative work behavior, while the relationship between algorithmic performance appraisal intensity and perceived professional autonomy was not statistically significant.

**Table 2:** Descriptive statistics and correlations in Study 2.

Variable	Cronbach’s *α*	CR	AVE	*M*	SD	1	2	3
1. Algorithmic performance appraisal intensity	0.848	0.873	0.581	3.74	1.32	0.762		
2. Perceived professional autonomy	0.819	0.838	0.563	3.80	1.49	−0.111	0.750	
3. Innovative work behavior	0.857	0.882	0.556	3.64	1.34	−0.057	0.226^***^	0.746

*N* = 287. ^***^p < 0.001.

#### 3.2.4 Results

Based on the measurement model results, we tested the mediating role of perceived professional autonomy. As shown in Table 3, after controlling for gender, age, occupational role, work experience, educational level, professional title, and hospital type, algorithmic performance appraisal intensity was not significantly related to perceived professional autonomy, *b* = −0.133, SE = 0.069, *t* = −1.926, *p* = 0.055. Therefore, H1 was not supported in Study 2. Perceived professional autonomy positively predicted innovative work behavior, *b* = 0.192, SE = 0.051, *t* = 3.790, *p* < 0.001, supporting H2. The direct effect of algorithmic performance appraisal intensity was not significant, *b* = −0.051, SE = 0.058, *t* = −0.874, *p* = 0.383, and its total effect was also non-significant, *b* = −0.076, SE = 0.059, *t* = −1.289, *p* = 0.198.

**Table 3:** Control-adjusted mediation test results in Study 2.

Model	Dependent variable	Predictor	*b*	SE	*t*	*p*	*R* ^2^
Model 1	Perceived professional autonomy	Algorithmic performance appraisal intensity	−0.133	0.069	−1.926	0.055	0.024
Model 2	Innovative work behavior	Algorithmic performance appraisal intensity	−0.051	0.058	−0.874	0.383	0.146
		Perceived professional autonomy	0.192	0.051	3.790	<0.001	
Model 3	Innovative work behavior	Algorithmic performance appraisal intensity, total effect	−0.076	0.059	−1.289	0.198	0.102

Indirect effect test		
Path	Indirect effect	95% bootstrap CI
Algorithmic performance appraisal intensity → Perceived professional autonomy → Innovative work behavior	−0.025	[−0.058, 0.0005]

*N* = 287. All models controlled for gender, age, occupational role, work experience, educational level, professional title, and hospital type. Full coefficients for all covariates are reported in Supplementary Table S2.

The control-adjusted indirect effect through perceived professional autonomy was −0.025, with a 95% bootstrap confidence interval of [−0.058, 0.0005]. Because the confidence interval included zero, H3 was not supported. The inclusion of the covariates did not materially alter the focal conclusions.

Overall, Study 2 supported H2 but not H1 or H3. Study 3 therefore examined the relationships using time-separated multisource data and tested the role of algorithmic transparency.

### 3.3 Study 3

#### 3.3.1 Research design and sample

Study 3 further examined whether algorithmic transparency moderates the relationship between algorithmic performance appraisal intensity and perceived professional autonomy, and whether this conditional process extends to innovative work behavior. Building on Study 2, we adopted a three wave, multisource matched design to reduce common method bias and improve the objectivity of the outcome measure. At T1, employees reported algorithmic performance appraisal intensity, algorithmic transparency, and control variables. About 3 weeks later, at T2, they reported perceived professional autonomy. After another 3 weeks, at T3, their direct supervisors rated their innovative work behavior.

The sample consisted of medical professionals and their direct supervisors from several general and specialized hospitals. Employees included physicians, nurses, and medical technicians, while supervisors were mainly department heads, head nurses, and other managers with direct evaluation responsibilities. Employees were required to have worked in their current positions for at least 6 months and to clearly identify their direct supervisors. Supervisors were required to be familiar with the employees’ daily work performance.

At T1, 360 employee questionnaires were distributed. After excluding invalid responses, 321 valid employee responses were retained. At T2, 289 valid employee responses were successfully matched. At T3, supervisors rated employees’ innovative work behavior, and 273 valid matched cases were obtained after removing incomplete or unmatched cases. The sample varied in gender, job type, age, work experience, educational level, professional title, and hospital type.

#### 3.3.2 Measures and analytical strategy

Study 3 measured algorithmic performance appraisal intensity, algorithmic transparency, perceived professional autonomy, innovative work behavior, and control variables. All items used a seven point Likert scale, ranging from 1, strongly disagree, to 7, strongly agree.

Algorithmic performance appraisal intensity was measured using the same five-item scale as in Study 2. The complete item wording and literature basis are reported in Supplementary Table S4.

Algorithmic transparency was measured using four items capturing employees’ understanding of the data sources, evaluation standards, calculation logic, and feedback basis of the hospital performance evaluation system. Sample items included “I clearly understand what data the hospital performance system mainly uses for evaluation,” “The scoring logic of the performance evaluation system is clear to employees,” and “I can understand how performance feedback results are generated.”

Perceived professional autonomy was measured using items adapted from Breaugh’s work autonomy scales and the Work Design Questionnaire (Breaugh, 1985, 1999; Morgeson and Humphrey, 2006). The items captured medical professionals’ perceived control over professional judgment, work methods, and problem handling.

Innovative work behavior was measured using items adapted from Scott and Bruce’s (1994) scale and was rated by direct supervisors at T3. Using supervisor ratings helped reduce social desirability bias associated with self-reports.

Gender, age, occupational role, work experience, educational level, professional title, and hospital type were included as covariates in all regression and conditional process analyses. Categorical variables were dummy coded. Algorithmic performance appraisal intensity and algorithmic transparency were mean-centered before constructing the interaction term. Table 4 reports the focal coefficients from the control-adjusted models, while the complete coefficients for all covariates are reported in Supplementary Table S3. Conditional indirect effects were estimated using 20,000 bootstrap samples.

**Table 4:** Control-adjusted moderation and outcome regression results in Study 3.

Model	Dependent variable	Predictor	*b*	SE	*t*	*p*	*R* ^2^
Model 1	Perceived professional autonomy	Algorithmic performance appraisal intensity	−0.189	0.062	−3.064	0.002	0.116
		Algorithmic transparency	0.150	0.059	2.559	0.011	
		Interaction term	0.046	0.044	1.038	0.300	
Model 2	Supervisor-rated innovative work behavior	Algorithmic performance appraisal intensity	−0.053	0.058	−0.912	0.363	0.080
		Algorithmic transparency	0.096	0.054	1.758	0.080	
		Perceived professional autonomy	0.126	0.057	2.212	0.028	

*N* = 273. All models controlled for gender, age, occupational role, work experience, educational level, professional title, and hospital type. Full coefficients for all covariates are reported in Supplementary Table S3.

#### 3.3.3 Results

Before testing the hypotheses, we assessed the internal consistency of the core variables. Cronbach’s alpha values were 0.860 for algorithmic performance appraisal intensity, 0.819 for algorithmic transparency, 0.809 for perceived professional autonomy, and 0.820 for supervisor rated innovative work behavior, indicating good reliability.

As shown in Table 5, algorithmic performance appraisal intensity was negatively related to perceived professional autonomy, *r* = −0.218, *p* < 0.001. Algorithmic transparency was positively related to perceived professional autonomy, *r* = 0.185, *p* = 0.002. Perceived professional autonomy was positively related to supervisor rated innovative work behavior, *r* = 0.186, *p* = 0.002. Algorithmic transparency was also positively related to supervisor rated innovative work behavior, *r* = 0.160, *p* = 0.008, whereas algorithmic performance appraisal intensity was not significantly related to supervisor-rated innovative work behavior, *r* = −0.115, *p* = 0.058.

**Table 5:** Descriptive statistics and correlations in Study 3.

Variable	Cronbach’s *α*	*M*	SD	1	2	3	4
1. Algorithmic performance appraisal intensity	0.860	3.70	1.28	–			
2. Algorithmic transparency	0.819	3.96	1.24	−0.130^*^	–		
3. Perceived professional autonomy	0.809	3.77	1.36	−0.218^***^	0.185^**^	–	
4. Supervisor rated innovative work behavior	0.820	3.64	1.24	−0.115^†^	0.160^**^	0.186^**^	–

^***^p < 0.001, ^**^p < 0.01, ^*^p < 0.05, ^†^*p* < 0.10.

After controlling for gender, age, occupational role, work experience, educational level, professional title, and hospital type, algorithmic performance appraisal intensity remained negatively related to perceived professional autonomy, *b* = −0.189, SE = 0.062, *t* = −3.064, *p* = 0.002. Algorithmic transparency was positively related to professional autonomy, *b* = 0.150, SE = 0.059, *t* = 2.559, *p* = 0.011. However, the interaction between appraisal intensity and transparency was not statistically significant, *b* = 0.046, SE = 0.044, *t* = 1.038, *p* = 0.300. Therefore, H1 was supported in Study 3, whereas H4 was not supported.

Perceived professional autonomy positively predicted supervisor-rated innovative work behavior, *b* = 0.126, SE = 0.057, *t* = 2.212, *p* = 0.028, supporting H2. Neither algorithmic performance appraisal intensity, *b* = −0.053, SE = 0.058, *t* = −0.912, *p* = 0.363, nor algorithmic transparency, *b* = 0.096, SE = 0.054, *t* = 1.758, *p* = 0.080, significantly predicted innovative work behavior after the covariates were included.

The conditional indirect effect was significant at low algorithmic transparency, effect = −0.032, 95% CI [−0.074, −0.001], but not at high algorithmic transparency, effect = −0.016, 95% CI [−0.055, 0.012]. However, the index of moderated mediation was not statistically significant, index = 0.0058, 95% CI [−0.0073, 0.0228]. Therefore, H5 was not supported.

Overall, Study 3 supported H1 and H2 but not H4 or H5. The inclusion of the covariates did not alter these hypothesis decisions. Algorithmic transparency remained positively related to professional autonomy, but its relationship with supervisor-rated innovative work behavior did not reach statistical significance after the covariates were included.

## 4 Discussion

### 4.1 Results and contributions

Across the three studies, the proposed model received partial support. H1 was supported in Studies 1 and 3 but not in Study 2, H2 was supported in Studies 2 and 3, and H3–H5 were not supported. The findings therefore provide stronger evidence for the separate relationships among algorithmic performance appraisal, professional autonomy, and innovative work behavior than for the proposed mediation and moderation processes.

Study 1 showed that an appraisal arrangement dominated by system-generated results, continuous data tracking, and rule-based comparison produced lower perceived professional autonomy than an arrangement in which supervisors retained greater contextual appraisal discretion. This finding is consistent with research suggesting that algorithmic management can alter work processes and performance evaluation practices and may weaken employees’ perceived autonomy in non-platform workplaces (Nilsson et al., 2025; Rani et al., 2024). The result suggests that increasing reliance on algorithmic outputs while reducing the role of contextual human judgment may weaken medical professionals’ perceived autonomy.

However, this relationship was not statistically significant in Study 2 and re-emerged in Study 3. Actual hospital appraisal systems are embedded in managerial practices, professional routines, and informal forms of discretion. Medical professionals may also adapt to established appraisal systems over time. These factors may help explain why the autonomy-related effect was less consistent across the field studies.

Studies 2 and 3 consistently showed that professional autonomy was positively associated with innovative work behavior. This finding is consistent with healthcare research linking innovation to discretionary space, role flexibility, and autonomy at work (Kessel et al., 2012; Baig et al., 2022). Recent nursing research likewise shows that professional autonomy is positively associated with innovative behavior (Lu et al., 2025).

Nevertheless, professional autonomy did not statistically mediate the relationship between algorithmic performance appraisal intensity and innovative work behavior. One possible explanation is that professional autonomy is a relatively proximal psychological state, whereas innovative work behavior requires not only discretion but also opportunities, resources, managerial approval, and tolerance for risk. In hospital settings, the promotion and implementation of new ideas may be constrained by team approval, clinical safety requirements, professional norms, and resource availability (Kessel et al., 2012; Baig et al., 2022). Moreover, the relationship between appraisal intensity and professional autonomy was not statistically significant in Study 2, which weakened the first stage of the proposed indirect process. Professional autonomy may therefore support innovative behavior without consistently transmitting the effect of algorithmic appraisal.

Study 3 showed that algorithmic transparency was positively associated with professional autonomy. However, it did not significantly moderate the relationship between appraisal intensity and autonomy, and its relationship with supervisor-rated innovative work behavior was not statistically significant after the covariates were included. These findings suggest that transparency is related to employees’ autonomy-related experience, but they do not provide evidence that transparency protects employees from the consequences of intensive algorithmic appraisal.

Taken together, the three studies are better interpreted as a reflective assessment of the proposed relationships than as cumulative confirmation of the full model. The findings suggest that algorithmic performance appraisal may constrain professional autonomy and that professional autonomy is relevant to innovative work behavior, but they do not establish the proposed mediation or buffering processes.

The findings offer three relatively conservative contributions. First, the study extends research on algorithmic management and performance appraisal to hospital settings, where standardized employee evaluation must coexist with professional judgment and contextual discretion (Rani et al., 2024). Second, Studies 2 and 3 provide consistent evidence that professional autonomy is positively associated with innovative work behavior among medical professionals, reinforcing previous healthcare research on the importance of discretion and agency for innovation (Kessel et al., 2012; Lu et al., 2025). Third, the findings clarify the role of algorithmic transparency. Transparency was positively associated with professional autonomy, but it did not significantly moderate the relationship between appraisal intensity and autonomy, and the index of moderated mediation was not significant. Its relationship with supervisor-rated innovative work behavior was also non-significant after the covariates were included. Transparency should therefore be understood primarily as being associated with employees’ autonomy-related experience rather than as a demonstrated protective mechanism.

### 4.2 Managerial implications

This study suggests that when hospitals promote digital governance and performance reform, they should not treat algorithmic performance appraisal only as a technical tool for improving efficiency and optimizing resource allocation. For medical professionals, the way performance evaluation is conducted affects their experience of professional judgment space and work agency, which in turn relates to innovative work behavior. Therefore, when designing digital performance evaluation systems, hospitals should not only focus on indicator completeness, system accuracy, and timely feedback. They should also consider whether the evaluation approach excessively compresses medical professionals’ autonomy. If performance governance relies too heavily on automated scoring, continuous comparison, and strong rule based feedback, medical professionals may gradually adjust passively around indicators rather than act proactively based on professional judgment. Such a model may improve short term controllability, but it may not support long term innovation capacity.

Hospitals should therefore avoid pushing algorithmic performance appraisal toward a single logic of quantitative reinforcement. Instead, they should pay more attention to the balance between performance systems and professional governance. Digital systems can improve information integration and reduce arbitrary evaluation, but medical work is highly complex, and many important professional values cannot be reduced to calculable indicators. Performance systems should not become the only basis for evaluation. They should be combined with departmental discussion, professional review, and managerial judgment, so that digital evaluation plays a reference and support role rather than mechanically defining professional work (Table 6).

**Table 6:** Conditional indirect effects and index of moderated mediation in Study 3.

Test	Effect	95% bootstrap CI
Conditional indirect effect at low algorithmic transparency	−0.032	[−0.074, −0.001]
Conditional indirect effect at high algorithmic transparency	−0.016	[−0.055, 0.012]
Index of moderated mediation	0.0058	[−0.0073, 0.0228]

This study also shows that perceived professional autonomy has a relatively stable positive relationship with innovative work behavior. This means that if hospitals want to improve medical professionals’ willingness to improve work practices and engage in innovation, they should not rely only on external incentives or higher evaluation intensity. They also need to maintain professionals’ sense of agency over their work through the organizational environment. For medical professionals, continuous improvement and proactive innovation are often supported by the belief that they still have space to propose new solutions, adjust work methods, and make judgments based on professional knowledge. Therefore, when promoting performance governance, hospitals should also build a more supportive professional environment. They can increase professional discussion mechanisms, improve channels for feedback on improvement suggestions, and develop cross department collaboration platforms. In this way, medical professionals are not only evaluated, but also able to participate in optimizing work processes and evaluation standards.

Algorithmic transparency was positively associated with professional autonomy, but it did not significantly buffer the relationship between appraisal intensity and professional autonomy or predict supervisor-rated innovative work behavior after the covariates were included. Hospitals should nevertheless make performance appraisal systems understandable by clearly explaining their data sources, evaluation logic, and feedback mechanisms. Such information may reduce uncertainty and allow employees to understand how appraisal outcomes are generated. However, the present findings do not indicate that transparency alone offsets the autonomy-related consequences of intensive algorithmic appraisal. Hospitals should therefore combine information disclosure with opportunities for contextual explanation, professional review, and human judgment.

Finally, from a talent management perspective, high level medical professionals are not merely objects to be evaluated by performance systems. They are also important sources of knowledge accumulation, process improvement, and service innovation in hospitals. If digital governance is mainly organized around short term performance visibility while ignoring its long term effects on professional autonomy and innovation potential, hospitals may strengthen control while weakening the professional vitality they most need to preserve. Future hospital governance should therefore pay more attention to the coordination between digital performance governance and professional talent development, and seek a more reasonable balance between governance efficiency and professional autonomy.

### 4.3 Limitations and future research

This study has several limitations. First, although the multi study design improves the robustness of the findings, the samples were mainly drawn from Chinese hospital settings. The external applicability of the conclusions should therefore be further examined across different countries, healthcare systems, and hospital types. Second, this study focused mainly on perceived professional autonomy as the psychological mechanism. In real organizational contexts, the effects of algorithmic performance appraisal on innovative work behavior may also operate through other pathways, such as procedural fairness, performance anxiety, or perceived organizational support. The current model is therefore relatively simplified. Although the study conceptually distinguishes algorithmic appraisal intensity from general performance pressure, performance pressure was not directly measured as a competing explanatory mechanism. Future research should measure both constructs simultaneously to determine whether algorithmic features explain autonomy beyond the effects of demanding targets, workload, and appraisal consequences. In Study 1, the manipulation varied both the reliance on algorithmically generated outputs and the relative role of human judgment. These two influences therefore cannot be separated within the current experimental design. Future research could use a factorial design that independently manipulates algorithmic reliance and human involvement, or retain the same level of human oversight while varying only the weight assigned to algorithm-generated scores. Third, algorithmic transparency was positively associated with professional autonomy, but its relationship with supervisor-rated innovative work behavior was not statistically significant after the covariates were included. The proposed moderation and moderated mediation effects were also not supported, indicating that the role of transparency requires further clarification. The differences across the three studies also indicate that the proposed model should be viewed as a theory-guided and reflective framework that requires further validation across organizational settings. Fourth, although supervisor ratings reduced common method bias in Study 3, supervisors may not have observed all aspects of employees’ daily innovative behavior, particularly informal idea generation, peer-level problem solving, and improvement efforts occurring outside their direct supervision. Future research could combine supervisor ratings with employee self-reports, peer assessments, and objective records of implemented improvements.

Future research can proceed in three directions. First, scholars can test the present model in different cultural and institutional contexts to improve the generalizability of the findings. Second, future studies can introduce additional mediating mechanisms and boundary conditions to build a more complete explanatory framework and reveal more deeply how algorithmic performance appraisal affects medical professionals’ behavioral responses. Third, future research can combine longitudinal data, experimental interventions, or richer multisource data designs to strengthen causal identification and examine more carefully the differentiated effects of algorithmic governance across occupational groups and organizational levels.

## 5 Conclusion

This study provides partial support for the proposed model. Algorithmic performance appraisal intensity was negatively related to professional autonomy in Studies 1 and 3, and professional autonomy consistently predicted innovative work behavior. However, the proposed mediation, moderation, and moderated mediation effects were not supported. The cross-study findings therefore support a reflective, rather than fully confirmatory, interpretation of the model, and algorithmic transparency should not be interpreted as a demonstrated protective mechanism.

## Data Availability

The raw data supporting the conclusions of this article will be made available by the authors, without undue reservation.
