# Supplementary material for: Algorithmic performance appraisal intensity in hospitals: implications for professional autonomy and innovative work behavior
Source: Front Psychol. 2026 Jul 29;17:1886547. doi: 10.3389/fpsyg.2026.1886547 (PMC13461422; doi:10.3389/fpsyg.2026.1886547)
Supplement: Supplementary file 1 [file Supplementary_file_1.docx]

Supplementary Table S1 Control-Adjusted Regression Results in Study 1

| **Predictor** | **Professional autonomy** |
| --- | --- |
| Male | −0.347 (0.173)* |
| Age | −0.050 (0.036) |
| Nurse | −0.037 (0.208) |
| Medical technician | −0.075 (0.256) |
| Medical postgraduate trainee | 0.298 (0.257) |
| Work experience | 0.038 (0.037) |
| High appraisal intensity | −1.110 (0.168)*** |
| R² | .190 |
| Adjusted R² | .166 |
| N | 240 |

Note. Entries are unstandardized coefficients, with standard errors in parentheses. Female and doctor are the reference categories. * p < .05; *** p < .001.

Supplementary Table S2 Control-Adjusted Regression Results in Study 2

| **Predictor** | **M1: Autonomy** | **M2: IWB** | **M3: IWB total effect** |
| --- | --- | --- | --- |
| Male | 0.081 (0.193) | 0.099 (0.162) | 0.114 (0.166) |
| Age | −0.023 (0.032) | −0.025 (0.027) | −0.029 (0.027) |
| Nurse | −0.064 (0.197) | −0.180 (0.165) | −0.192 (0.169) |
| Medical technician | −0.282 (0.276) | −0.180 (0.232) | −0.234 (0.237) |
| Work experience | 0.024 (0.031) | 0.031 (0.026) | 0.035 (0.026) |
| Master’s degree | −0.125 (0.196) | −0.634 (0.165)*** | −0.658 (0.168)*** |
| Doctorate degree | 0.199 (0.346) | −0.818 (0.291)** | −0.780 (0.297)** |
| Intermediate title | −0.004 (0.199) | −0.386 (0.167)* | −0.387 (0.171)* |
| Senior title | −0.178 (0.278) | −0.066 (0.233) | −0.100 (0.239) |
| Specialty hospital | 0.020 (0.209) | −0.133 (0.175) | −0.129 (0.179) |
| Appraisal intensity | −0.133 (0.069) | −0.051 (0.058) | −0.076 (0.059) |
| Professional autonomy | — | 0.192 (0.051)*** | — |
| R² | .024 | .146 | .102 |
| Adjusted R² | −.015 | .109 | .066 |
| N | 287 | 287 | 287 |

Note. Entries are unstandardized coefficients, with standard errors in parentheses. IWB = innovative work behavior. Female, doctor, bachelor’s degree, junior professional title, and general hospital are the reference categories. * p < .05; ** p < .01; *** p < .001.

Note. Entries are unstandardized coefficients, with standard errors in parentheses. IWB = innovative work behavior. Female, doctor, bachelor’s degree, junior professional title, and general hospital are the reference categories. * p < .05; ** p < .01; *** p < .001.

Supplementary Table S3 Control-Adjusted Regression Results in Study 3

| **Predictor** | **M1: Autonomy** | **M2: Supervisor-rated IWB** |
| --- | --- | --- |
| Male | 0.004 (0.173) | 0.022 (0.158) |
| Age | 0.016 (0.034) | 0.025 (0.031) |
| Nurse | −0.041 (0.185) | 0.126 (0.170) |
| Medical technician | −0.497 (0.242)* | 0.113 (0.223) |
| Work experience | −0.006 (0.033) | −0.020 (0.030) |
| Master’s degree | 0.058 (0.177) | 0.062 (0.162) |
| Doctorate degree | 0.597 (0.337) | 0.210 (0.310) |
| Intermediate title | −0.221 (0.181) | −0.364 (0.166)* |
| Senior title | 0.027 (0.263) | −0.076 (0.241) |
| Specialty hospital | 0.160 (0.186) | −0.126 (0.169) |
| Appraisal intensity | −0.189 (0.062)** | −0.053 (0.058) |
| Algorithmic transparency | 0.150 (0.059)* | 0.096 (0.054) |
| Appraisal intensity × transparency | 0.046 (0.044) | — |
| Professional autonomy | — | 0.126 (0.057)* |
| R² | .116 | .080 |
| Adjusted R² | .072 | .034 |
| N | 273 | 273 |

Note. Entries are unstandardized coefficients, with standard errors in parentheses. IWB = innovative work behavior. Female, doctor, bachelor’s degree, junior professional title, and general hospital are the reference categories. Appraisal intensity and transparency were mean-centered. * p < .05; ** p < .01.

Supplementary Table S4. Items Measuring Algorithmic Performance Appraisal Intensity

| **Item** | **Item wording** |
| --- | --- |
| APEI1 | The hospital continuously uses digital systems to collect and track data about my individual work performance. |
| APEI2 | The performance appraisal system automatically compares my work-performance data with predefined standards or relevant benchmarks. |
| APEI3 | My performance is evaluated largely according to predefined rules embedded in the digital system. |
| APEI4 | The digital system automatically generates scores, rankings, or feedback about my work performance. |
| APEI5 | System-generated results play an important role in the hospital’s final appraisal of my individual work performance. |
